# RIPK3 and Caspase-1/11 Are Necessary for Optimal Antigen-Specific CD8 T Cell Response Elicited by Genetically Modified *Listeria monocytogenes*

**DOI:** 10.3389/fimmu.2020.00536

**Published:** 2020-04-09

**Authors:** Aamir Rana, Felipe Campos de Almeida, Henry A. Paico Montero, Maryanne M. Gonzales Carazas, Karina R. Bortoluci, Subash Sad, Gustavo P. Amarante-Mendes

**Affiliations:** ^1^Instituto de Ciências Biomédicas, Universidade de São Paulo, São Paulo, Brazil; ^2^Instituto de Investigação em Imunologia, Instituto Nacional de Ciência e Tecnologia (INCT), São Paulo, Brazil; ^3^Departamento de Ciências Biológicas, Centro de Terapia Celular e Molecular (CTC-Mol), Universidade Federal de São Paulo, São Paulo, Brazil; ^4^Department of Biochemistry, Microbiology and Immunology, Faculty of Medicine, University of Ottawa, Ottawa, ON, Canada

**Keywords:** Effector CD8^+^ T cells, *Listeria monocytogenes*, ovalbumin, RIPK3, Casp-1/11

## Abstract

Efficient induction of effector and long-term protective antigen-specific CD8^+^ T memory response by vaccination is essential to eliminate malignant and pathogen-infected cells. Intracellular infectious bacteria, including *Listeria monocytogenes*, have been considered potent vectors to carry multiple therapeutic proteins and generate antigen-specific CD8^+^ T cell responses. Although the role of molecules involved in inflammatory cell death pathways, such as necroptosis (RIPK3-mediated) and pyroptosis (Caspase-1/11-mediated), as effectors of immune response against intracellular bacteria are relatively well understood, their contribution to the adjuvant effect of recombinant bacterial vectors in the context of antigen-specific CD8^+^ T cell response remained obscure. Therefore, we evaluated the impact of RIPK3 and Caspase-1/11 (Casp-1/11) individual and combined deficiencies on the modulation of antigen-specific CD8^+^ T cell response during vaccination of mice with ovalbumin*-*expressing *L. monocytogenes* (LM-OVA). We observed that Casp-1/11 but not RIPK3 deficiency negatively impacts the capacity of mice to clear LM-OVA. Importantly, both RIPK3 and Casp-1/11 are necessary for optimal LM-OVA-mediated antigen-specific CD8^+^ T cell response, as measured by *in vivo* antigen-specific CD8^+^ T cell proliferation, target cell elimination, and cytokine production. Furthermore, Casp-1/11 and Casp-1/11/RIPK3 combined deficiencies restrict the early initiation of antigen-specific CD8^+^ T cell memory response. Taken together, our findings demonstrate that RIPK3 and Casp-1/11 influence the quality of CD8^+^ T cell responses induced by recombinant *L. monocytogenes* vectors.

## Introduction

CD8^+^ cytotoxic T lymphocytes (CTLs) are key cells in host immune defense against pathogens and cancer (1). CTLs exert their activation, clonal expansion, and differentiation upon TCR engagement with its cognate antigen in the context of MHC-class I on the surface of antigen-presenting cells (APCs) (2, 3). Importantly, the interaction of CD28 on CTLs with CD80 (B7-1) or CD86 (B7-2) avoids CTL anergy (4–6). Once activated and differentiated, CTLs produce cytokines such as IFN-γ, TNF-α, and IL-2 to enhance effector CD8^+^ T cell differentiation and kill target cells via FASL or by the combination of perforin and granzymes (7, 8).

*Listeria monocytogenes* (LM) is a gram-positive intracellular foodborne bacterial pathogen that causes listeriosis in pregnant women, newborn babies, and immune-compromised individuals (9). Preferential accumulation of LM into the cytoplasm of infected cells potentiates the presentation of LM-expressing antigens through MHC-I restricted pathway for CD8^+^ T cell priming (10–13). This results in a strong antigen-specific CD8^+^ T cell response (2), which peaks at 7–10 days after primary infection (14, 15). Because of the ability of LM to potently induce CTL response (2), recombinant LM carrying single or multiple therapeutic proteins have been proposed as vaccination vectors against cancer or other unrelated chronic infectious diseases (16–18). Indeed, over the years, more than 30 clinical trials testing 10 different attenuated LM cancer vaccines alone and/or in combination to different drugs have been initiated (19). Importantly, LM-based vaccines have been shown to display mild side effects, such as transient fever, chills, vomiting, nausea, and hypotension (20–23). Only a few patients developed systemic listeriosis, which could be properly controlled by antibiotics (24, 25). Therefore, as LM-based vaccines hold promise, it is important to develop a better understanding of immune response triggered by recombinant LM in preclinical settings (19).

Caspase-1 and caspase-11 activation in the context of inflammasomes assembly results in the cleavage of Gasdermin D (GsdmD)—the pyroptosis executioner (26, 27). Activation of inflammasomes such as NLRP3, NLRC4, and AIM2 by LM activates Casp-1/11 to trigger pyroptosis and IL-1β and IL-18 secretion, thus amplifying the inflammatory process (28). In addition, it was reported that LM activates RIPK3, which further phosphorylates mixed lineage kinase domain-like protein (MLKL), but MLKL activation does not result in plasma membrane disruption and necroptosis. Interestingly enough, phosphorylated MLKL directly binds with LM to prevent its cytosolic replication (29). Although LM infection activates RIPK3-MLKL without inducing necroptosis (29) and triggers Casp-1/11 activation through inflammasomes (28), the direct role of RIPK3 and Casp-1/11 in the generation and modulation of antigen-specific CD8^+^ T cell response after LM infection remained obscure.

It is conceivable that the level of immune response against vaccination vectors has a direct impact on the effector and memory response against the recombinant protein engineered in such vectors. Therefore, genetic deficiencies that may impact host immunity against *listeria* likely alter the efficiency of *listeria*-based vaccination strategy. The current study was designed to investigate the role of RIPK3 and Casp-1/11 in the induction and modulation of antigen-specific effector and memory CD8^+^ T cell response generated by LM-OVA. Our results demonstrate that RIPK3 and Casp-1/11 deficiencies directly impact the clearance of LM-OVA infection. Most importantly, both RIPK3 and Casp-1/11 deficiencies limit antigen-specific CD8^+^ T cell effector and early memory response against the recombinant OVA protein.

## Materials and Methods

### Mice

C57BL/6 RIPK3^–/–^ mice were previously generated by Newton et al. (30) and generously provided by Vishva Dixit (Genentech, Inc., United States). C57BL/6 Casp-1/11^–/–^ mice were kindly provided by Richard Flavell (Yale University, United States). Six- to 8-week-old WT, RIPK3^–/–^, Casp-1/11^–/–^, and Casp-1/11^–/–^/RIPK3^–/–^ double-deficient male mice were used as experimental controls or infected groups. OT-I mice CD45.1^+^45.2^+^ were generated by mating OT-I males (45.1^–^45.2^+^) with B6.SJL (CD45.1^+^45.2^–^) females, as previously described (31). All mouse experiments were performed at the animal facilities of Institute of Biomedical Sciences, University of São Paulo, and of University of Ottawa under the guidelines of the Ethics Committee on Animal Use, University of São Paulo and Canadian Council on Animal Care (CCAC), respectively.

### Bacteria and Infection

Recombinant LM strain (10403S) expressing ovalbumin (OVA) has been described previously (32). Bacteria were grown and stored as described previously (33). For infections, frozen stocks of LM-OVA were thawed and serially diluted in 0.9% NaCl. All the experimental mice were infected or not with 10^3^CFU of LM-OVA in 100 μl of 0.9% NaCl via the lateral tail vein.

### Viral Strain and Immunization

Recombinant human adenovirus-expressing ovalbumin vector (rhAd5.OVA) was kindly provided by José Ronnie C. Vasconcelos (Universidade Federal de São Paulo, Brazil). WT, RIPK3^–/–^, and Casp-1/11^–/–^ mice were immunized or not intramuscularly with 2 × 10^6^ PFU of rhAd5.OVA in a total volume of 100 μl of phosphate-buffered saline (PBS) (50 μl injected into the left and 50 μl into the right *Tibialis anterior* muscle).

### Bacterial Burden per Spleen

Spleens from all infected mice at 3 and 7 days post-infection were harvested individually and kept in RPMI-1640 medium (Life Technologies, Burlington, Ontario, Canada). Single-cell suspension was prepared by tweezing each spleen separately between the frosted ends of two sterile glass slides. CFU/spleen was determined by plating 10-fold serial dilutions of single cell suspension from individual spleen on BHI-Streptomycin agar plates.

### Assessment of Antigen-Specific CD8^+^ T Cell Population

All experimental groups were infected or not with 10^3^ LM-OVA for 7 days. At 7 days post-infection, spleens were harvested, processed to a single-cell suspension, and stained individually with anti-mouse CD8 antibody (BD Biosciences, 563898) and H2-K^*b*^-SIINFEKL Dextramer (Immudex, Copenhagen, Denmark, JD2163) according to manufacturer’s instruction. Samples were analyzed by FACS using BD FACSCelesta^TM^ (BD, Mountain View, CA, United States). Each sample was analyzed independently by using the gating strategy shown in Supplementary Figure S1. Finally, the frequency of CD8^+^ H2-K^*b*^–SIINFEKL^+^ population of each sample was evaluated separately using FlowJo v10 workspace.

### *In vivo* Proliferation of Antigen-Specific OT-I CD8^+^ T Cells and Adoptive Transfer

*In vivo* proliferation of OT-I CD8^+^ T cells (CD45.1^+^ and CD45.2^+^) was performed to evaluate the differences in the priming and proliferation pattern of the OT-I CD8^+^ T cells in WT and knockout (KO) mice. OT-I splenocytes were labeled with 5 μM of Cell tracer Violet (CTV) (CellTrace^TM^ Violet Cell Proliferation Kit, Invitrogen, C34557) and 10^7^ cells in 100 μl of un-supplemented RPMI medium were adoptively transferred by retro-orbital sinus in each mouse. After 1 h, mice were infected with LM-OVA, while control groups remained uninfected. Four days later, the spleens of recipient mice were collected and processed individually to make single-cell suspension. Splenocytes from each mouse were labeled independently with anti-CD8 (BioLegend, 100707) for 30 min in PBS containing 1% bovine serum albumin (BSA). The reduction of CTV staining in OT-I cells, as a measure of proliferation, was analyzed by flow cytometry using BD FACSCelesta^TM^ (BD, Mountain View, CA). Each sample was analyzed independently by using the gating strategy shown in Supplementary Figure S2 and the frequency of dividing OT-I cells obtained using the FlowJo v10 workspace.

### *In vivo* Cytotoxic Assay

*In vivo* cytotoxicity was performed as previously described (34) with slight modifications and optimization. Briefly, after 7 days post-infection, spleens from WT donor mice were harvested and processed for single cell suspension by tweezing each spleen between the frosted ends of two sterile glass slides. Cells were counted and divided equally into four populations and marked separately with two different concentrations of both Carboxyfluresceine succinimidyl Ester (CellTrace^TM^ CFSE Cell Proliferation Kit, Invitrogen, United States, C34554) and CTV; CFSE^*High*^ (10 μM), CFSE^*Low*^ (1 μM), CTV^*High*^ (10 μM), and CTV^*Low*^ (1 μM). CFSE^*High*^ cells were pulsed with 10 nM of OVA_257__–__264_ (SIINFEKL) peptide (InvivoGen, vac-sin) while the control CFSE^*Low*^ remained un-pulsed. CTV^*High*^ and CTV^*Low*^ cells were pulsed with 0.1 and 0.001 nM of OVA_257__–__264_ peptide, respectively. All four populations of cells were washed and mixed in 1:1:1:1 ratio. A total of 4 × 10^7^ cells in 100 μl of un-supplemented RPMI medium were inoculated in each infected and control experimental mouse by retro-orbital venous sinus. After 16–20 h, spleen from each mouse was excised, processed to obtain single-cell suspension, and analyzed individually by FACS using BD LSRFortessa^TM^ (BD, Mountain View, CA, United States). The gating strategy is shown in Supplementary Figure S3. Finally, the frequencies of live cells were analyzed using FlowJo v10 workspace.

### Enumeration of Antigen-Specific IFN-γ and TNF-α-Producing CD8^+^ T Cells

Enumeration of antigen-specific IFN-γ and TNF-α-producing CD8^+^ T cells was done by ELISPOT assay. Initially, 96-well nitrocellulose plate (Multiscreen HA Millipore) was coated with 60 μl per well of sterile 1 × PBS containing 10 ng/ml of mouse anti-IFNγ capture antibody (BD Bioscience, 551216). A separate 96-well nitrocellulose plate was coated with 100 μl per well of sterile 1 × PBS containing 1:100 dilution of mouse anti-TNF-α (BD Biosciences, 51-26732E). Both plates were incubated overnight at room temperature. After incubation, plates were washed three times with 100 μl of RPMI medium under sterile conditions. Subsequently, plates were blocked by adding 100 μl per well of RPMI-10% FBS (fetal bovine serum) medium for 2 h at 37°C. 10^6^ responder cells from each experimental mouse were separately added in anti-IFN-γ and anti-TNF-α Ab-coated ELISPOT plates, with 3 × 10^6^ feeder cells (from non-infected WT mice) and pulsed with 10 μM OVA_257__–__264_ peptide. The culture was established in RPMI medium supplemented with 1% NEAs (non-essential amino acids) (Gibco), 1% L-Glut (L-Glutamine) (Gibco), 0.1% β-mercaptoethanol (Gibco), 1% sodium pyruvate (Gibco), 1% Pen-Strep (penicillin–streptomycin) (Gibco), 1% vitamins (MEM vitamin solution) (Gibco), 10% FBS (Gibco), and 5 ng/ml recombinant mouse IL-2 (ThermoFisher, 701080) for 24 h and 36 h (IFN-γ and TNF-α, respectively) at 37°C with 5% CO_2_. After incubation plates were washed three times with PBS containing 0.05% of Tween-20 (Fisher BioReagents) (PBS-T). IFN-γ-specific plate was incubated with 100 μl per well of Biotin Rat Anti-Mouse IFN-γ (BD Biosciences, 554410) at a final concentration of 20 ng/ml in PBS-T at 4°C for overnight. TNF-α-specific plate was incubated with 100 μl per well of biotinylated anti-mouse TNF monoclonal antibody (BD Biosciences, 51-26731E) at a 1:100 dilution in PBS-T at 4°C overnight. The next day, the plates were washed five times with PBS-T and three times with 1 × PBS. Subsequently, 100 μl per well of PBS-T containing streptavidin-HRP complex (BD Bioscience, 554066) was added at a 1:800 dilution and incubated at room temperature for 2 h. Plates were washed three times with PBS-T and five times with 1 × PBS, respectively. Spots were visualized by using 3-Amino-9-ethylcarbazole (AEC) substrate (BD Biosciences, 551951) as per manufacturer’s instruction and washed with distilled water. The plates were dried at room temperature. The spots were quantified by ELISPOT reader (AID ELR06).

### Intracellular Cytokines Staining

For intracellular staining, spleens from infected and non-infected mice were obtained individually after 7 days of infection. A total of 4 million splenocytes from each mouse were cultured in RPMI medium supplemented with 1% NEAs, 1% L-Glut, 0.1% β-mercaptoethanol, 1% sodium pyruvate, 1% Pen-Strep, 1% vitamins (MEM vitamin solution), 10% FBS, 5 ng/ml recombinant mouse IL-2, 2 μg/ml purified Na/LE Hamster Anti-mouse CD28 (BD, Pharmingen^TM^, 553294), 1% brefeldin A (Biolegend, 420601), and 1% monensin (Biolegend, 420701). Cells were pulsed with 10 μg of OVA_257__–__264_ peptide and incubated for 8 h at 37°C with 5% CO_2_. To stain the surface marker CD107a (LAMP1), anti CD107a (BioLegend, 121609) was added in the complete medium. After 8 h of incubation, cell surface markers were stained with H2-K^*b*^-SIINFEKL Dextramer (Immudex, Copenhagen, Denmark, JD2163) and anti-CD8 (BD Biosciences, 551162). Intracellular staining of IFN-γ (BD Biosciences, 563376) and TNF-α (BD Biosciences, 554418) was done by using BD Cytofix/Cytoprem kit (BD Biosciences, 554714), as per manufacturer’s instruction. Cells were harvested and analyzed independently, by FACS using BD LSRFortessa^TM^ (BD, Mountain View, CA, United States). Gate strategy to observe the surface expression of CD8^+^ LAMP1^+^ is shown in Supplementary Figure S4. Gate strategy to observe intracellular expression of IFN-γ and TNF-α expressing CD8^+^ H2-K^*b*^–SIINFEKL^+^ is shown in Supplementary Figure S5. Finally, FlowJo v10 workspace was used to evaluate the frequencies of LAMP1-, IFN-γ-, and TNF-α-positive cells.

### Assessment of CD8^+^ T Memory Cell Differentiation

Splenocytes from OT-1 (CD45.1^+^ and CD45.2^+^) mice were obtained, processed, and inoculated (10^7^ cells) in each experimental mouse by retro-orbital venous sinus. After 1 h, mice were infected or not with 10^3^CFU of LM-OVA. At 7 days post-inoculation/infection, splenocytes from infected or non-infected mice were obtained, processed, and stained with anti-CD8 (eBioscience, 48-0081-82), anti-CD45.1 (eBioscience, 12-0453-82), anti-CD127 (eBioscience, 17-1271-82), and KLRG1 (eBioscience, 11-5893-80). Cells were harvested and analyzed independently by FACS using BD LSRFortessa^TM^ (BD, Mountain View, CA, United States), using the gating strategy shown in Supplementary Figure S6. Finally, FlowJo v10 workspace was used to evaluate the frequencies of CD127- and KLRG1-positive cells.

### Statistical Analysis

Statistical analysis was performed by using GraphPad Prism version 5 (GraphPad Software Company Incorporation). Values were expressed as mean ± SEM. Statistical significance was determined by using two-way ANOVA followed by Bonferroni post-tests. Statistical differences were considered significant when the *P*-value was <0.05.

## Results

### RIPK3 and Casp-1/11 Deficiencies Differentially Impair Host Ability to Control *Listeria* Infection

**FIGURE 1:**
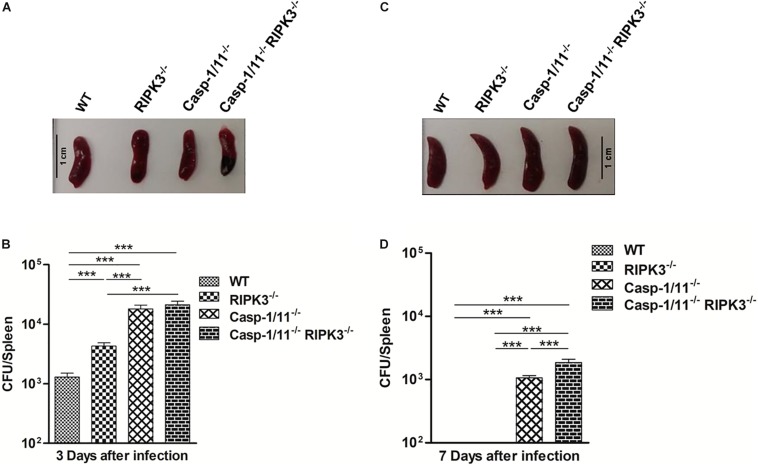
Casp-1/11 but not RIPK3 is necessary for the host ability to clear LM-OVA infection. All the experimental mice were infected for 10^3^ CFU of LM-OVA for 3 and 7 days, respectively. Spleen size was measured at day 3 and day 7 post-infection. Spleen from each infected mice was harvested and processed individually to make single-cell suspensions. CFU/spleen was determined by plating 10-fold serial dilutions of single-cell suspension from each individual mouse on BHI-Streptomycin agar plates. **(A,C)** Representative images of the spleen size and **(B,D)** bacterial burden were measured in infected mice at 3 **(A,B)** and 7 **(C,D)** days after LM-OVA infection. Results expressed as the mean of five individual mice per group and are representative of three independent experiments. Statistical analysis was performed by using two-way ANOVA followed by Bonferroni post-tests. ***p < 0.001.

First, we evaluated the impact of individual or combined RIPK3 and Casp-1/11 deficiencies on the ability of C57Bl/6 mice to handle recombinant LM-OVA infection. We observed no difference in the size of the spleens from WT, RIPK3^–/–^, Casp-1/11^–/–^, and Casp-1/11^–/–^/RIPK3^–/–^ DKO (double knock-out) mice at day 3 post-infection (peak of infection in the spleen) with LM-OVA (Figure 1A). However, at this time point, bacterial burden in spleen was significantly higher in RIPK3^–/–^, Casp-1/11^–/–^, and Casp-1/11^–/–^/RIPK3^–/–^ double deficient in comparison to WT mice, suggesting that both RIPK3 and Casp-1/11 are important to control the early phase of listeria infection (Figure 1B). Interestingly, at day 7 post-infection (time of resolution), the spleen size was augmented in all RIPK3^–/–^, Casp-1/11^–/–^, and Casp-1/11^–/–^/RIPK3^–/–^ DKO mice in comparison to WT (Figure 1C). Most importantly, at this time point, bacterial burden was only detected in Casp-1/11^–/–^ and Casp-1/11^–/–^/RIPK3^–/–^ DKO mice, suggesting that Casp-1/11 but not RIPK3 is essential to clear LM-OVA infection (Figure 1D).

### Casp-1/11 Deficiency Interferes With Antigen-Specific CD8^+^ T Cell Expansion in Response to LM-OVA

**FIGURE 2:**
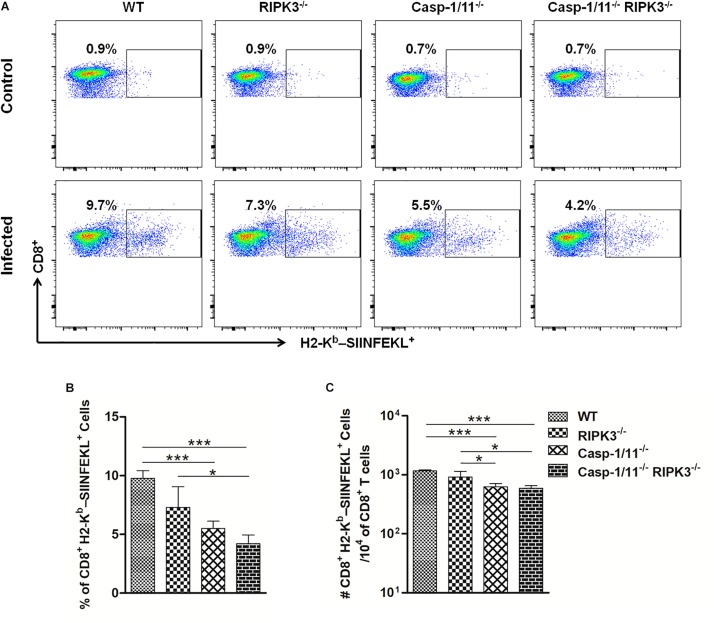
Casp-1/11 deficiency impairs *in vivo* antigen-specific CD8^+^ T cell expansion in response to LM-OVA. **(A,B)** Frequency and **(C)** total number of OVA_257__–__264_ (SIINFEKL)-specific CD8^+^ T cells in the spleens of infected mice at 7 days after LM-OVA vaccination, revealed by staining with anti-CD8 antibody and H2-K^*b*^-SIINFEKL dextramer. Results are expressed as **(A)** selected representative image per group or **(B,C)** representative bar graphic of three independent experiments showing the mean and standard error of five mice per group. Statistical analysis was performed by using two-way ANOVA followed by Bonferroni post-tests. *p < 0.5, ***p < 0.001.

To evaluate whether the ability to clear LM-OVA would influence the level of OVA-specific CD8^+^ T cell response, we used an MHC class I multimer technology to measure the population of OVA_257__–__264_ (SIINFEKL)-specific CD8^+^ T cells generated in response to LM-OVA vaccination. In comparison to WT mice, we observed significantly lower frequencies and absolute numbers of OVA_257__–__264_ (SIINFEKL)-specific CD8^+^ T cells in Casp-1/11^–/–^ and Casp-1/11^–/–^RIPK3^–/–^ DKO but not in RIPK3^–/–^ mice (Figures 2A–C), suggesting a positive correlation between the capacity of mice to clear LM-infection and the amplitude of OVA-specific CD8^+^ T cell proliferation.

### RIPK3 and/or Casp-1/11 Deficiencies Limit Antigen-Specific CD8^+^ T Cells Priming and Proliferation

**FIGURE 3:**
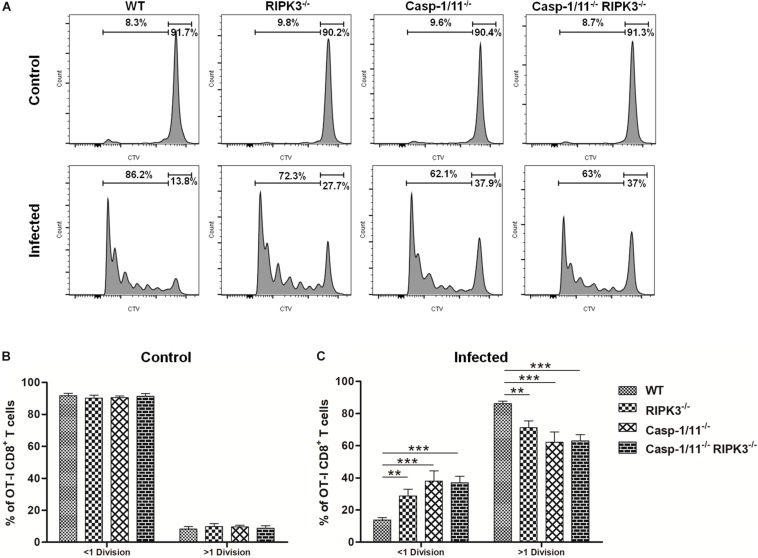
RIPK3 and/or Casp-1/11 deficiencies negatively affects OVA-specific CD8^+^ T cell priming and proliferation in response to LM-OVA. **(A)** Representative images of OT-I CD8^+^ T cells *in vivo* proliferation in non-infected and LM-OVA-infected WT, RIPK3^– /–^, Casp-1/11^– /–^, and Casp-1/11^– /–^/RIPK3^– /–^ mice. Frequencies of <1 division and >1 division OT-I CD8^+^ T cell populations in **(B)** non-infected controls and **(C)** LM-OVA-infected mice. **(B,C)** Results are expressed as means of five individual mice per group and are representative of three independent experiments. Statistical analysis was performed by using two-way ANOVA followed by Bonferroni post-tests. **p < 0.01, ***p < 0.001.

The reduced LM-OVA-triggered, OVA-specific CD8^+^ T cell numbers observed in the absence of Casp-1/11 (but not of RIPK3) could be the result of the impaired proliferation of these cells. To approach this question, we examined the *in vivo* proliferation of OT-I CD8^+^ T cells adoptively transferred to WT, RIPK3^–/–^, Casp-1/11^–/–^, or Casp-1/11^–/–^/RIPK3^–/–^ DKO mice at 96 h after vaccination with LM-OVA. As expected, we retrieved roughly the same frequency of OT-I CD8^+^ T cells in all non-infected mice strains, which means that the RIPK3 and/or Casp-1/11 deficiency does not affect the homeostatic proliferation or the survival of donor OT-I CD8^+^ T cells (Figures 3A,B). In contrast, compared with WT mice, we observed a significantly lower proliferation of OT-I CD8^+^ T cells in all RIPK3^–/–^, Casp-1/11^–/–^, and Casp-1/11^–/–^/RIPK3^–/–^ DKO mice in response to LM-OVA vaccination (Figures 3A,C), suggesting that the observed defect is extrinsic to the CD8 + T cells. Since the bacterial burden was high on day 3 in the RIPK3 and/or Casp-1/11 KO mice, this suggests that the impaired proliferation of CD8^+^ T cells in these mice is not due to poor antigenic levels. Accordingly, the frequency of non-dividing (>1 division) OT-I CD8^+^ T cell population remained significantly higher in RIPK3^–/–^, Casp-1/11^–/–^, and Casp-1/11^–/–^/RIPK3^–/–^ DKO mice (Figures 3A,C). Our results suggest that RIPK3 and Casp-1/11 are important for optimal priming and proliferation of antigen-specific CD8^+^ T cells in response to LM-OVA vaccination.

### RIPK3 and/or Casp-1/11 Deficiencies Impair OVA-Specific CD8^+^ T Cell Cytolytic Activity and Cytokine Production in Response to LM-OVA Vaccination

**FIGURE 4:**
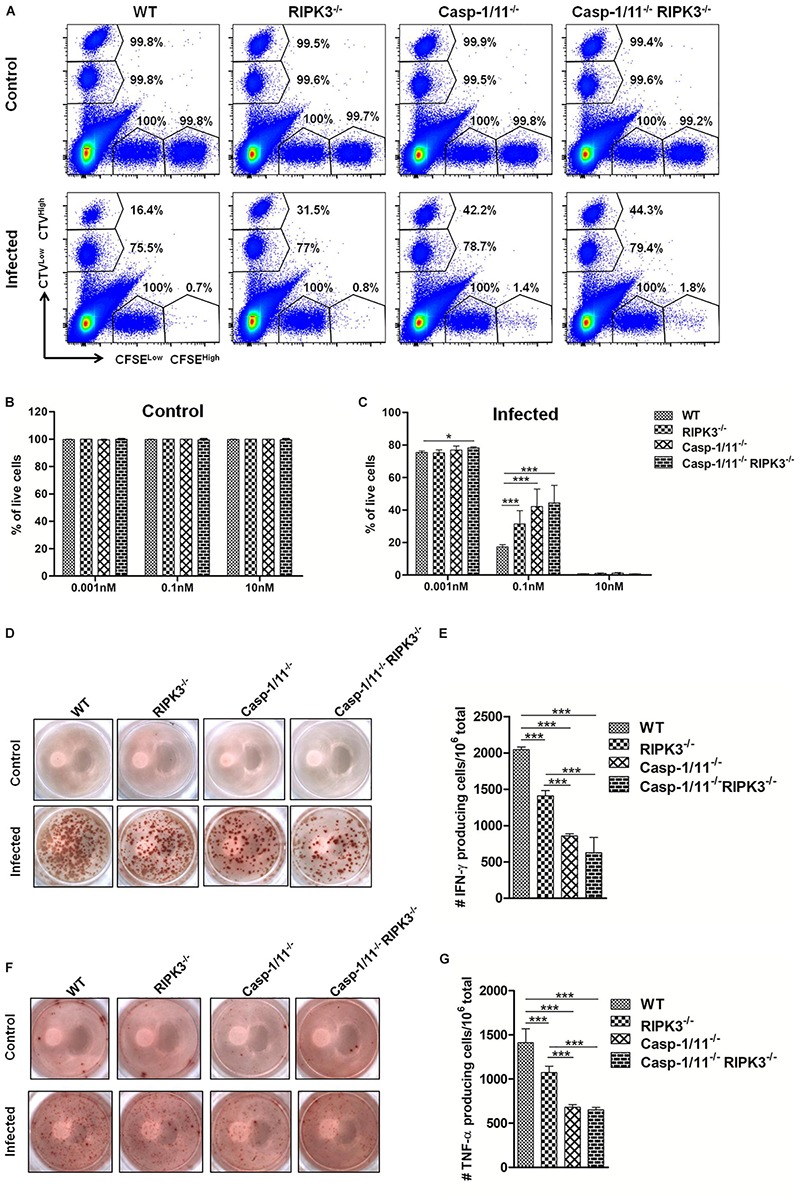
RIPK3 and/or Casp-1/11 deficiencies negatively affect *in vivo* antigen-specific CTL activity and cytokine production in response to LM-OVA. **(A)** *In vivo* elimination of target cells pulsed with high (10 nM) (CFSE^*High*^), intermediate (0.1 nM) (CTV^*High*^), and low (0.001 nM) (CTV^*Low*^) concentration of OVA_257__–__264_ peptide at 7 days post-infection with LM-OVA. Percentage of CFSE^*High*^, CFSE^*Low*^ (non-target cells), CTV^*High*^, and CTV^*Low*^ shows the frequency of remaining cells after CTL-mediated target cell elimination. Percentage of live target cells in **(B)** non-infected and **(C)** infected mice. **(D–G)** ELISPOT assay determined the frequency of OVA_257__–__264_ peptide-specific IFN-γ- or TNF-α-producing CD8^+^ T cells. **(A)** Flow cytometry density plots represent a single mouse from each experimental group. Numbers represent mean percentages of five mice/group. **(D,F)** ELISPOT images from a single mice/group are representative of five animals/group. **(B,C,E,G)** Bar graphs depict means and standard deviation of five individual mice per group. Experiments were repeated at least three times with similar results. Statistical analysis was performed by using two-way ANOVA followed by Bonferroni post-tests. *p < 0.05, ***p < 0.001.

**FIGURE 5:**
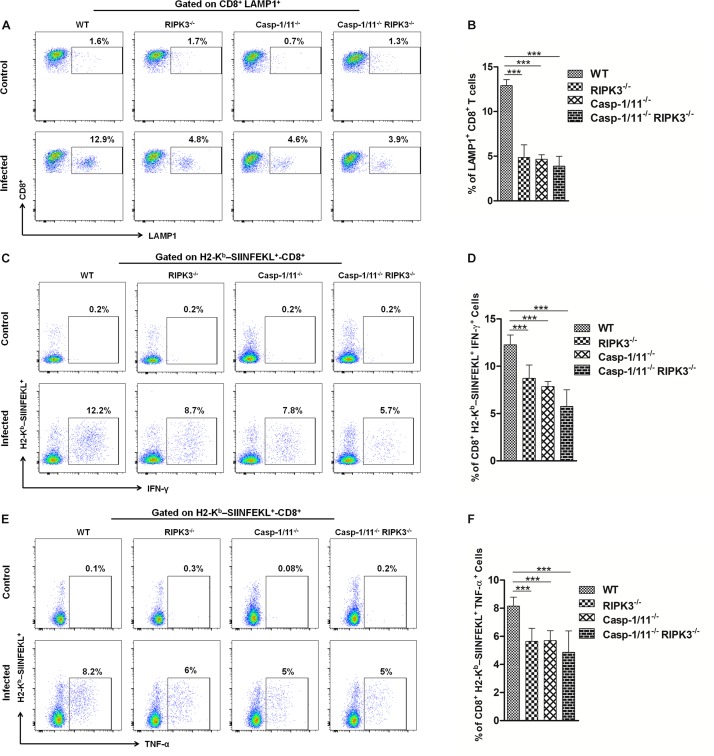
RIPK3 and/or Casp-1/11 deficiencies reduce antigen-specific CD8^+^ T cells degranulation and intracellular cytokine production in response to LM-OVA. **(A)** Representative images of frequency of LAMP-1-positive OVA-specific CD8^+^ T cells for LM-OVA-infected and non-infected (control) groups and the **(B)** frequency variation of LAMP-1^+^-CD8^+^ T cells observed on the LM-OVA-infected group. Immediately, representative image of intracellular H2-K^*b*^-SIINFEKL-specific **(C)** IFN-γ and **(E)** TNF-α-producing CD8^+^ T cell expression on control and infected groups. The frequency of these cell populations, H2-K^*b*^-SIINFEKL-specific **(D)** IFN-γ and **(F)** TNF-α-producing CD8^+^ T cells, after 7 days of infection with LM-OVA was schematized. Frequencies described in **(B,D,F)** bar graphs were representative of experiments done in triplicate, and the data depict mean ± SEM of five individual mice per group. Statistical analysis was performed by using two-way ANOVA followed by Bonferroni post-tests. ***p < 0.001.

Next, we analyzed the importance of RIP3K and Casp-1/11 for the functional profile of CD8^+^ T cells. First, we evaluated the cytolytic activity of OVA-specific CD8^+^ T cells at day 7 post-infection with LM-OVA. Syngeneic splenocytes (target cells) were pulsed with three different concentrations of OVA_257__–__264_ peptide, namely, high (10 nM), intermediate (0.1 nM), and low (0.001 nM) concentrations. As expected, we retrieved more than 99% of all target and non-target populations in control, non-infected mice (Figures 4A,B). A significant weakening in elimination of target cells pulsed with 0.1 nM of OVA_257__–__264_ peptide was observed in RIPK3^–/–^, Casp-1/11^–/–^, and Casp-1/11^–/–^/RIPK3^–/–^ DKO mice (Figures 4A,C), suggesting that at least for this particular concentration of cognate peptide (therefore avidity of CTL/target cell interaction may be critical), both RIPK3 and Casp-1/11 are important for optimal *in vivo* CTL effector response. Interestingly, we also observed a small reduction (yet significant) in target cell elimination at the lower peptide concentration (0.001 nM) in Casp-1/11^–/–^/RIPK3^–/–^ DKO mice (Figures 4A,C), suggesting that these proteins may act in concert to optimize the protective effect of LM-OVA vaccination. Further, we assessed the ability of OVA-specific CD8^+^ T cells to produce IFN-γ or TNF-α by ELISPOT and intracellular staining. A significant lower frequency of IFN-γ-producing (Figures 4D,E) and TNF-α-producing (Figures 4F,G) CD8^+^ T cells was observed in RIPK3^–/–^, Casp-1/11^–/–^, and Casp-1/11^–/–^/RIPK3^–/–^ mice. To determine the prerequisite of cytolytic activity of OVA-specific CD8^+^ T cells, we assessed the degranulation of OVA-specific CD8^+^ T cells by measuring the surface expression of CD107a, a lysosomal associated membrane protein (LAMP-1). RIPK3^–/–^ and Casp1-11^–/–^ deficiency resulted in a significant reduction in LAMP-1 expression (Figures 5A,B). Similarly, a significantly reduced expression of IFN-γ (Figures 5C,D) and TNF-α (Figures 5E,F) was observed in OVA-specific CD8^+^ T cells by intracellular staining. Taken together, our results indicate that the cytolytic activity and cytokine production of antigen-specific CD8^+^ T cells are promoted by RIPK3 and Casp1-11.

### Casp-1/11 and Casp-1/11/RIPK3 Combined Deficiencies Restrict OVA-Specific CD8^+^ T Memory Cell Differentiation

**FIGURE 6:**
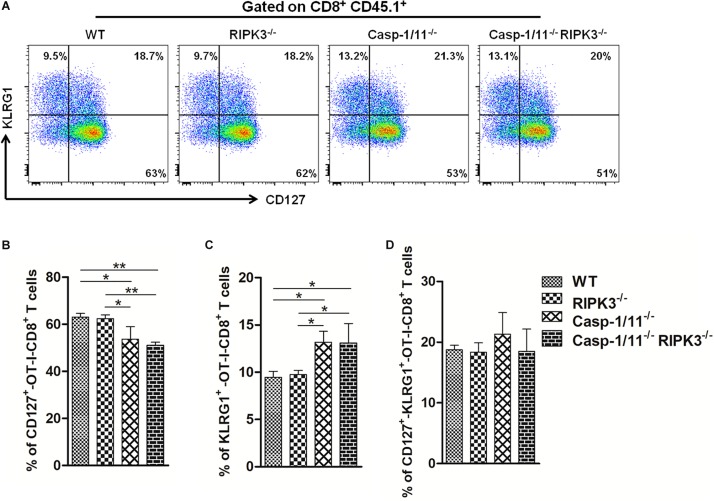
Casp-1/11 and Casp-1/11, RIPK-3 combined deficiencies restrict early OVA-specific CD8^+^ T memory cell response. **(A)** Surface expression of CD127 and KLRG1 on adoptively transferred CD8^+^ T cells after 7 days of infection was denoted by flow cytometry representative images. Frequency of **(B)** CD127, **(C)** KLRG1, and **(D)** CD127, KLRG1-positive OT-1 CD8^+^ T cells are expressed as means of five individual mice per group and are representative of three independent experiments. Statistical analysis was performed by using two-way ANOVA followed by Bonferroni post-tests. *p < 0.05, **p < 0.01.

To correlate the downgrading of OVA-specific CD8^+^ T cytolytic activity and cytokine production with early initiation of memory response cells, we evaluated the surface expression of memory precursor molecules (CD127^*high*^ KLRG1^*low*^) on adoptively transferred OT-I CD8^+^ T cells after 7 days of infection. Casp-1/11 but not RIP3K deficiency significantly reduced the expression of CD127 with an increase of KLRG1 expression on OT-I CD8^+^ T cells after 7 days of infection (Figures 6A–D). Thus, our results indicate that RIPK3 and Casp-1/11 differentially modulate the early differentiation of memory precursor OVA-specific CD8^+^ T cells.

## Discussion

An efficient immunity against cancer and pathogens involves specific recognition and removal of malignant or infected cells. Strategies aimed to elicit optimized effector CD8^+^ T cell response, in a way that single antigen-specific clones may present multiple effector functions and differentiate to long-lived memory T cells, have been proposed (35). Among such strategies, the use of intracellular infectious bacteria carrying single or multiple therapeutic proteins holds promise (36, 37). In fact, it has been previously reported that recombinant *L. monocytogenes* carrying the ovalbumin gene (LM-OVA) induces strong OVA-specific CD8^+^ T cell response and protects mice against B16-OVA melanoma cell line (38). Importantly, the role of RIPK3 and Casp-1/11, proteins known to be involved in the control of *L. monocytogenes* infection, on OVA-specific CD8^+^ T cell response after LM-OVA vaccination remained unexplored.

Recently, it has been reported that activation of RIPK3 during LM infection contributes to restrict intracellular replication of the parasite (29). Interestingly enough, LM-induced activation of RIPK3 results in phosphorylation of MLKL without inducing necroptosis (29), suggesting a necroptosis-independent role or RIPK3 in LM restriction. Here, we show that RIPK3 deficiency negatively impacts the control of LM-OVA burden only at the early stage (day 3) of infection. In contrast, at the peak of antigen-specific CD8^+^ T cell response (day 7), both WT and RIPK3-deficient mice were able to completely eliminate LM-OVA infection. We were not able to determine whether the effect of RIPK3 deficiency on the early control of LM-OVA was necroptosis-dependent or -independent. Regardless, although the impact of the absence of RIPK3 on the control of LM-OVA burden was mild and temporary, it significantly reduced the OVA-specific CD8^+^ T cell proliferation, cytolytic activity and cytokine production, suggesting a role for RIPK3 on the control of optimal adaptive immune responses following recombinant LM vaccination.

Similarly to RIPK3, it has been reported that deficiency of Casp-1/11 renders mice more susceptible to LM infection (39, 40). In agreement, we observed that Casp-1/11 deficiency also facilitates LM-OVA infection at both early (day 3) and late (day 7) stages of infection. Remarkably, the absence of Casp-1/11 had an even more profound negative effect on OVA-specific CD8^+^ T cell responses compared to the lack of RIPK3. Importantly, the combined RIPK3 and Casp-1/11 deficiencies did not result in synergistic or additive effects. Actually, Casp-1/11, RIPK3 double-deficient mice behaved similarly to Casp-1/11-deficient mice in every aspect investigated in our work. Therefore, our data suggest that RIPK3 and Casp-1/11 operate in the same functional pathway (see below) and that Casp-1/11 is dominant over RIPK3.

Importantly, the observed deficiency of OVA-specific CD8^+^ T cell response does not seem to be related to an intrinsic flaw of CD8^+^ T cells from RIPK3^–/–^ or Casp-1/11^–/–^ mice. *In vivo* proliferation of RIPK3/Casp-1/11-sufficient OT-I CD8^+^ T cells occurred normally in LM-OVA-infected WT but not on RIPK3-, Casp-1/11-, or Casp-1/11/RIPK3-deficient mice, suggesting that the observed deficiency is set at the level of the activation of CD8^+^ T cells by APCs.

Efficient activation and optimal expansion of effector CD8 + T cell response depend on LM intracellular burden and the level of infection was shown to impact the priming ability of infected APCs (41, 42). Despite the observed susceptibility of RIPK3- and/or Casp-1/11-deficient mice to LM, we were not able to formally establish a direct link between degree of pathogen clearance and the magnitude of antigen-specific CD8^+^ T cell responses. However, it is important to note that although RIPK3KO as well as WT mice were able to clear LM at day 7, all three KO strains were less efficient to handle LM at day 3, as compared to WT mice. Interestingly, our results show that the deficiency of CD8^+^ T cell activation in RIPK3KO is mild compared to the deficiency observed in Casp1/11 KO or in RIP3K/Casp1/11 DKO, which could be related to the degree of pathogen clearance by these mice. Nevertheless, it is still obscure whether the inefficient LM clearance by innate immune mechanisms is responsible for impaired CD8^+^ T cell response, or a delayed/ineffective adaptive CD8^+^ T cell immunity partially contributes to defective LM clearance, or both. In any case, it is reasonable to believe that the efficiency to clear LM is somehow linked to an optimal induction of CD8^+^ T cell response and both are regulated by RIPK3 and Casp-1/11.

Because the defect is apparently not intrinsic to CD8^+^ T cells, one should expect that macrophages and/or DCs, the cells that connect the innate to the adaptive immune system, would be the major targets of RIPK3 and Casp-1/11 deficiency. In this regard, interaction of deficient macrophages and/or DCs with LM could result in compromised antigen processing, cytokine production, expression of co-stimulatory molecules, etc. All these features could be the consequence of impaired intracellular signaling cascades (related or not to abnormal cell death) and could negatively influence CD8^+^ T cell activation. Studies to explore these possibilities are underway in our laboratory.

We also compared the role of RIPK3 and Casp-1/11 deficiencies on the induction of early memory antigen-specific CD8^+^ T cells during LM-OVA infection. Intriguingly, our data show that Casp-1/11 and Casp-1/11/RIPK3 double deficiency restricts initiation of early memory precursor antigen-specific CD8^+^ T cell response, as observed by the CD127^*high*^ KLRG1^*low*^ phenotype, while individual deficiency of RIPK3 does not affect the dynamic of memory precursor CD8^+^ T cell population. These results may suggest that Casp-1/11 but not RIPK3 are necessary to the proper generation and differentiation of memory CD8^+^ T cells in response to LM-OVA.

Interestingly, the combined deficiency of RIPK3 and Casp-1/11 allows survival of OVA-expressing *Salmonella typhimurium* (ST-OVA) in DCs and macrophages leading to antigen-specific CD8^+^ T cells that overexpress TIM3 and PD-1 (31). Furthermore, infection of double-deficient (RIPK3^–/–^ Casp-1/11^–/–^) mice with ST-OVA results in a higher frequency of IFN-γ-producing CD8^+^ T cells (31). In contrast, we found significantly reduced IFN-γ- or TNF-α-producing CD8 + T cells after LM-OVA infection in RIPK3- and/or Casp-1/11-deficient mice. Moreover, we found no significant differences in antigen-specific CD8^+^ T cell differentiation and effector response after immunization with recombinant human adenovirus expressing ovalbumin (rhAd5-OVA) (Supplementary Figure S7). Taken together, we suggest that RIPK3 and Casp-1/11 participate differently in antigen-specific effector and memory CD8^+^ T cell response generated by different recombinant live vector. Furthermore, our findings may help to optimize the immunotherapeutic potential of LM- or other live vector-based vaccination strategies.

## Data Availability Statement

The raw data supporting the conclusions of this article will be made available by the authors, without undue reservation, to any qualified researcher.

## Ethics Statement

The animal study was reviewed and approved by the Ethics Committee on Animal Use, University of São Paulo and Canadian Council on Animal Care (CCAC), Canada. Written informed consent was obtained from the owners for the participation of their animals in this study.

## Author Contributions

AR, KB, and GA-M designed the study. AR performed the experiments. AR, FC, HP, MG, SS, and GA-M analyzed and interpreted the data. AR, KB, and GA-M wrote the manuscript. GA-M, KB, and SS read and approved the final manuscript.

## Conflict of Interest

The authors declare that the research was conducted in the absence of any commercial or financial relationships that could be construed as a potential conflict of interest.
