# Supplementary material for: RIPK3 and Caspase-1/11 Are Necessary for Optimal Antigen-Specific CD8 T Cell Response Elicited by Genetically Modified Listeria monocytogenes
Source: Front Immunol. 2020 Apr 9;11:536. doi: 10.3389/fimmu.2020.00536 (PMC7160319; doi:10.3389/fimmu.2020.00536)
Supplement: Supplementary file 1 [file Data_Sheet_1.pdf]

Supplementary Material

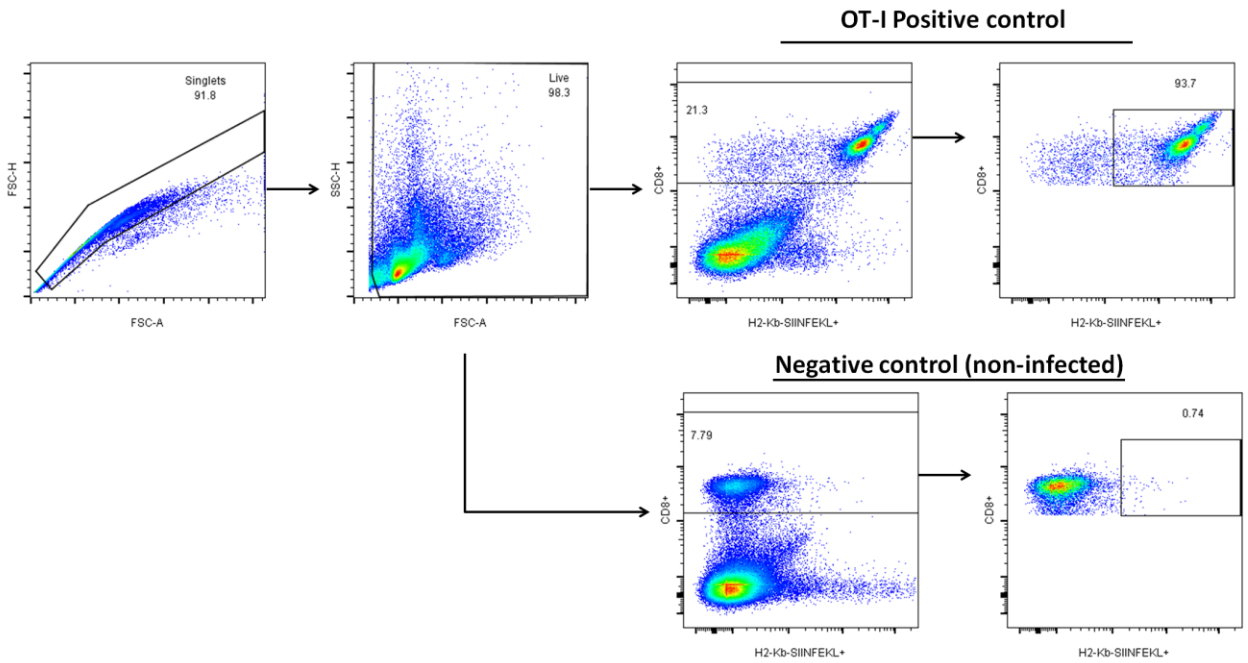

Supplementary figure 1: Gate strategy to evaluate CD8$^+$ H2-K^b–SIINFEKL$^+$ population. FSC-A x FSC-H (Forward Scattered-Area x Forward Scattered-Height) to exclude doublets, then FSC-A x SSC-H (Forward Scattered-Area x Side Scattered-Height) to exclude debris and finally H2-K^b–SIINFEKL$^+$ x CD8$^+$ to separate CD8$^+$ H2-K^b–SIINFEKL$^+$ population.

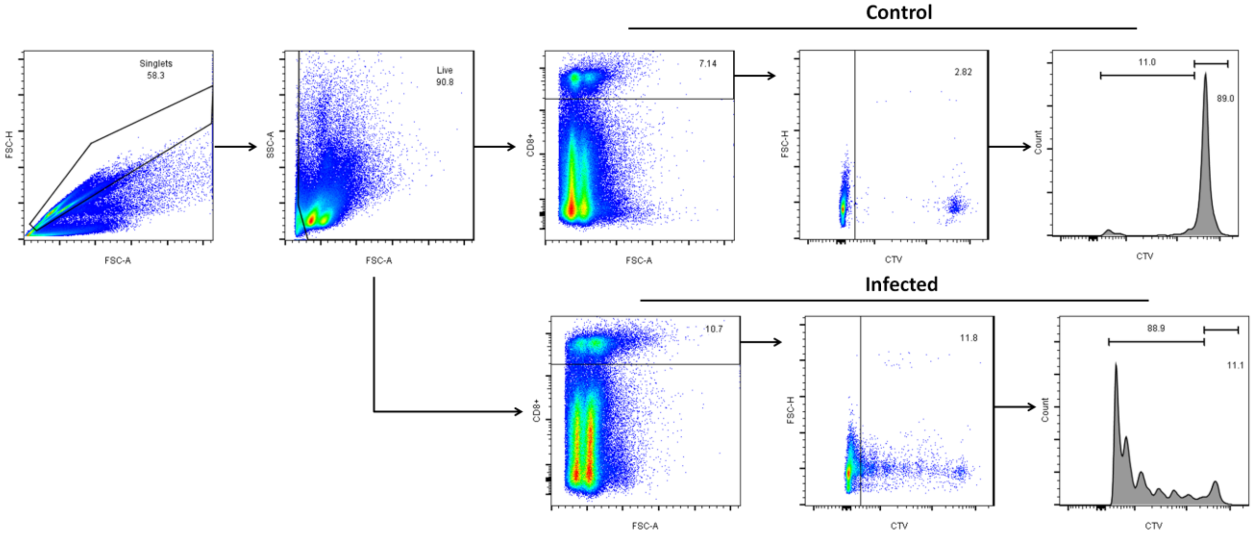

Supplementary figure 2: Gate strategy to evaluate *in vivo* proliferation of OT-I cells. FSC-A x FSC-H to exclude doublets, FSC-A x SSC-A (Forward Scattered-Area x Side Scattered-Area) to exclude debris, FSC-A x $CD8^+$ to separate $CD8^+$ T cell population, FSC-H x CTV to separate adoptively transferred OT-I $CD8^+$ cell population and finally CTV x Count to observe proliferation/division of OT-I CD8+ T cell population.

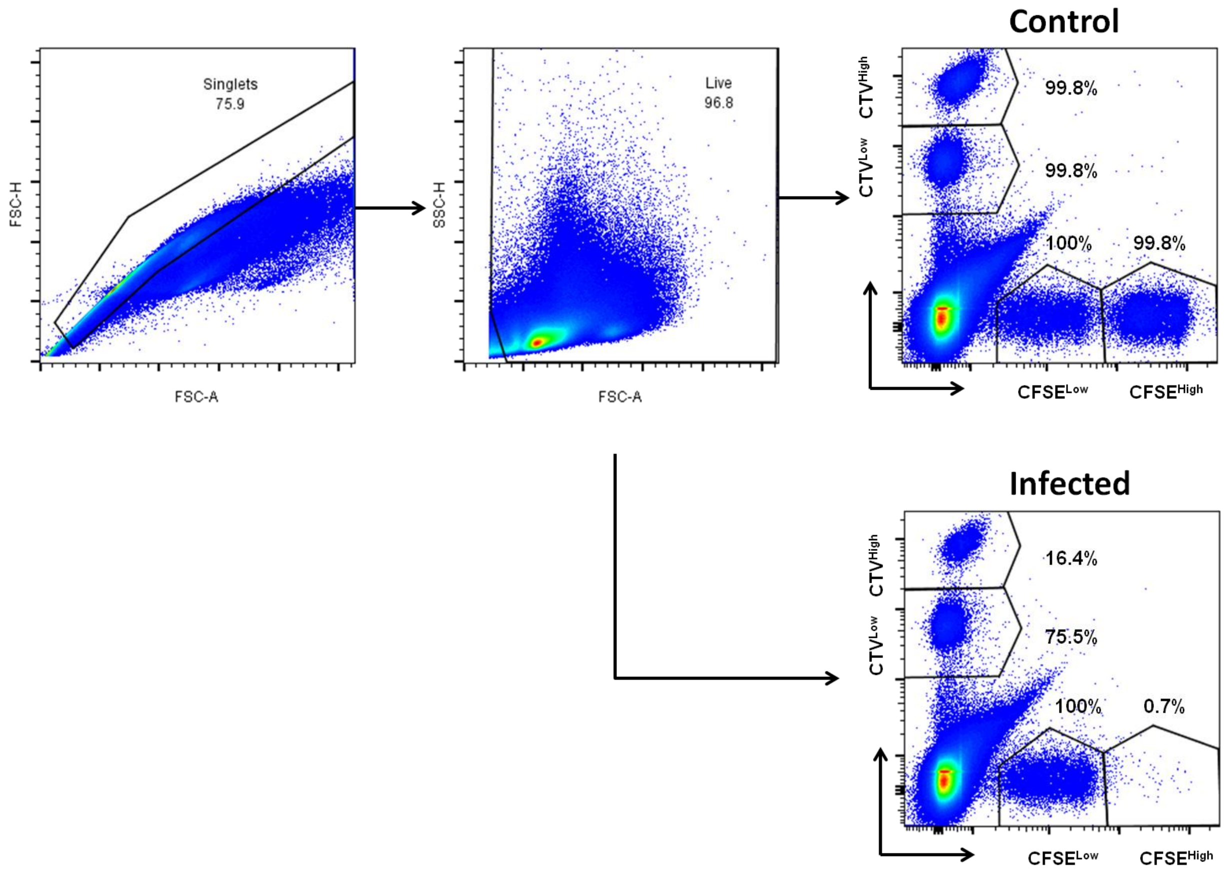

Supplementary figure 3: Gate strategy to evaluate in vivo target cell killing by CTLs. FSC-A x FSC-H to exclude doublets, FSC-A x SSC-H to exclude debris and finally CFSE^{High}, CFSE^{Low}, CTV^{High} and CTV^{Low} populations showing percentage of target cell killing by CTLs.

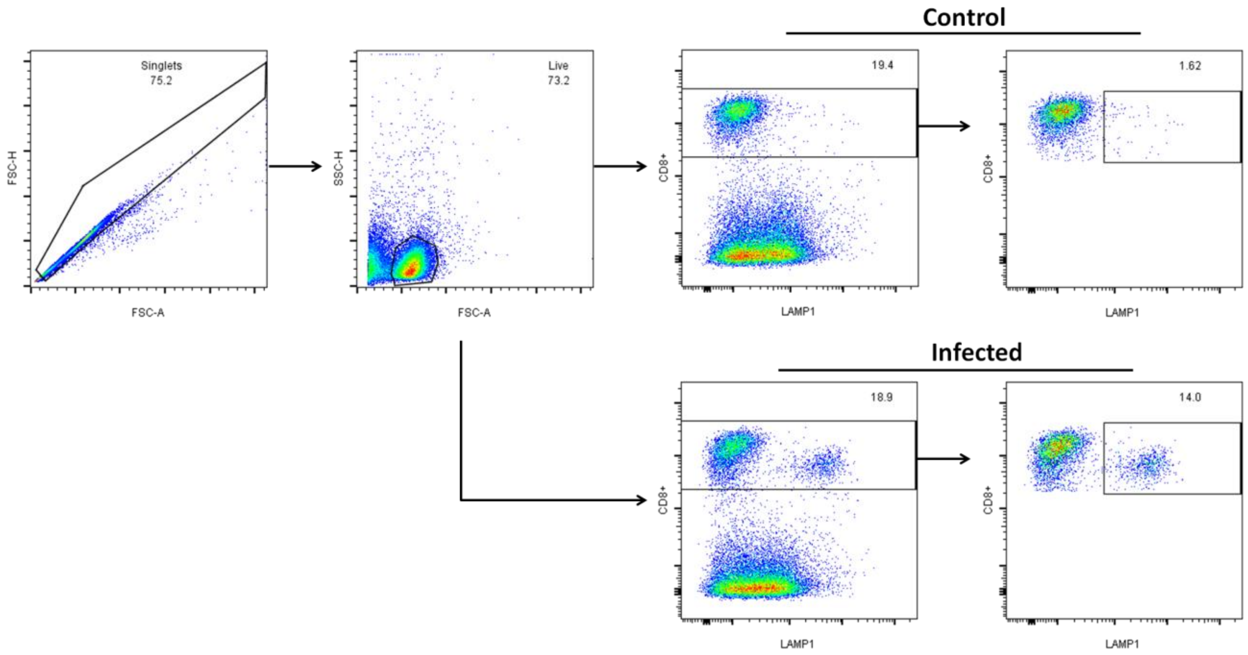

Supplementary figure 4: Gate strategy for CD8$^+$ LAMP1$^+$ population. FSC-A x FSC-H to exclude doublets, then FSC-A x SSC-H to exclude debris and finally LAMP1 x CD8$^+$ to separate CD8$^+$ LAMP1$^+$ population.

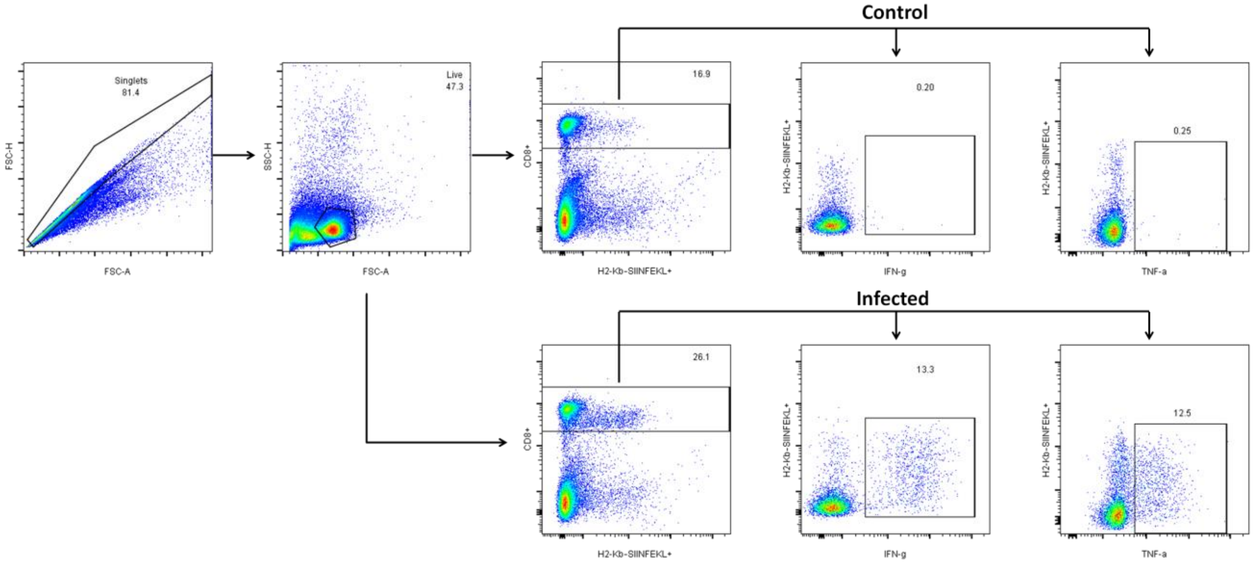

Supplementary figure 5: Gate strategy for IFN-γ and TNF-α expressing CD8$^+$ H2-K^b–SIINFEKL$^+$ population. FSC-A x FSC-H to exclude doublets, then FSC-A x SSC-H to exclude debris, H2-K^b–SIINFEKL$^+$ x CD8$^+$ to separate H2-K^b–SIINFEKL$^+$ x CD8$^+$ population, IFN-γ x H2-K^b–SIINFEKL$^+$ to separate IFN-γ$^+$ population and TNF-α x H2-K^b–SIINFEKL$^+$ to separate TNF-α$^+$ population.

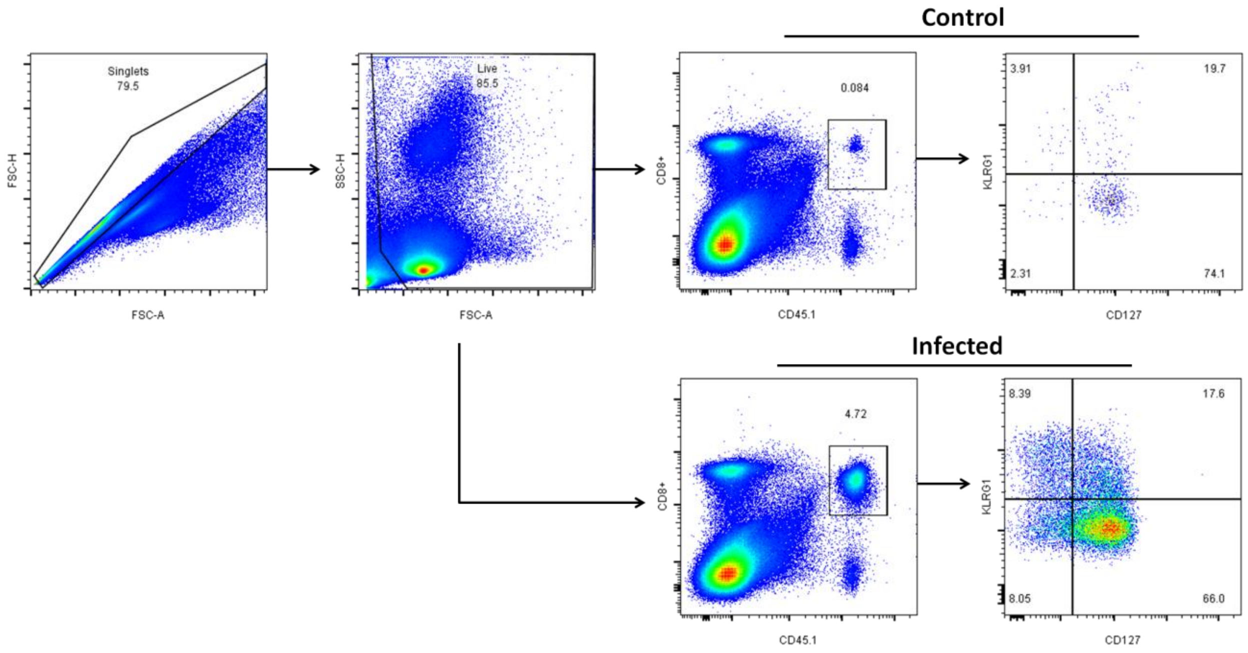

Supplementary figure 6: Gate strategy to evaluate KLRG1$^+$ CD127$^+$ OT-I CD8$^+$ (CD45.1 CD45.2$^+$) population. FSC-A x FSC-H to exclude doublets, then FSC-A x SSC-H to exclude debris, CD45.1 x CD8$^+$ to separate CD45.1$^+$ CD45.2$^+$ adoptively transferred CD8$^+$ OT-1 cells and finally CD127 x KLRG1 to observe the frequency of KLRG1 and CD127$^+$ adoptively transferred CD8+ OT-1 cells.

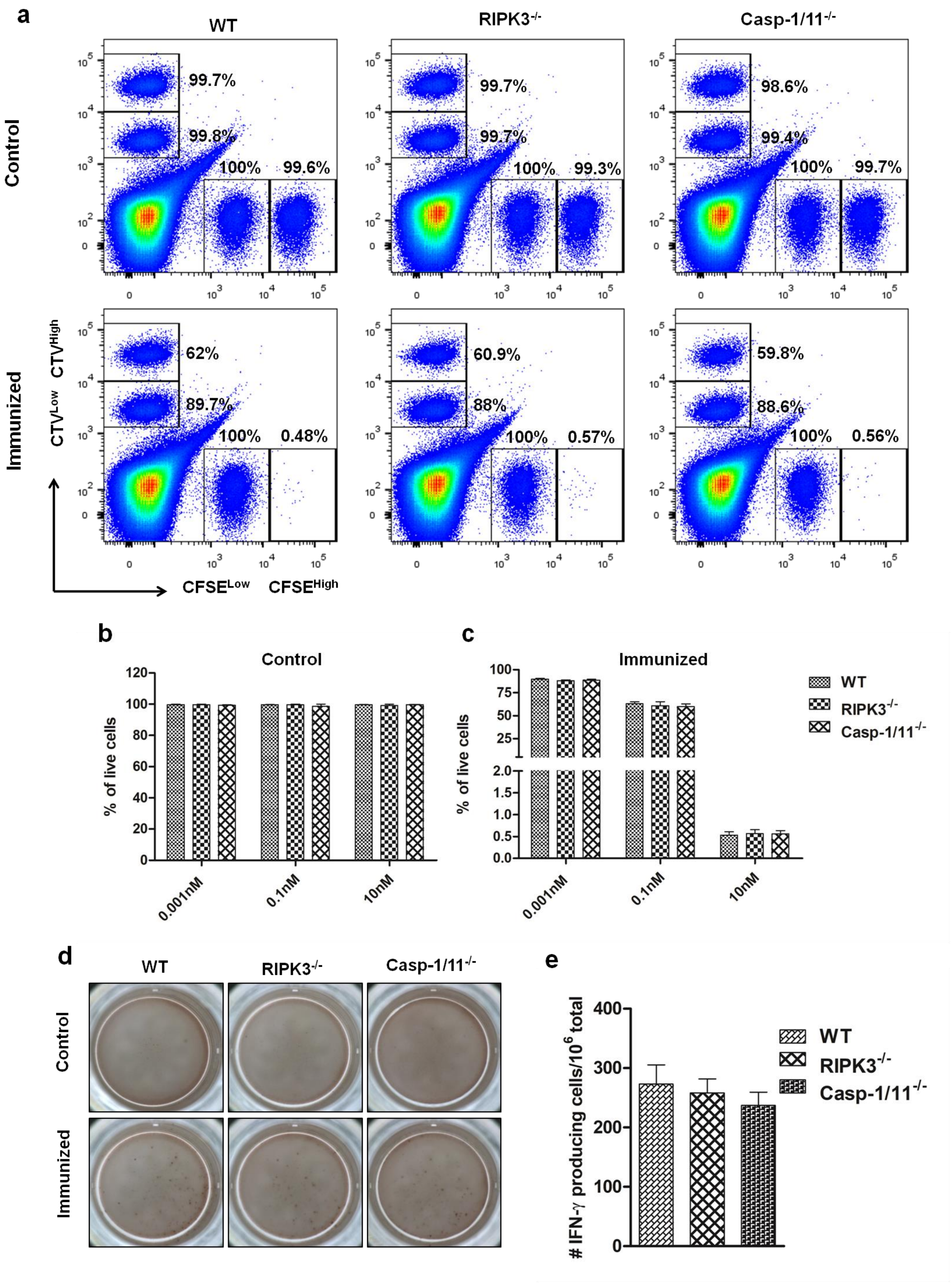
a
WT
RIPK3-/-
Casp-1/11-/-
Control
Immunized
99.7%
99.8%
100%
99.6%
99.7%
99.7%
100%
99.3%
98.6%
99.4%
100%
99.7%
62%
89.7%
100%
0.48%
60.9%
88%
100%
0.57%
59.8%
88.6%
100%
0.56%
CTV^Low CTV^High
CFSE^Low CFSE^High
b
Control
% of live cells
0.001nM
0.1nM
10nM
c
Immunized
% of live cells
0.001nM
0.1nM
10nM
WT
RIPK3-/-
Casp-1/11-/-
d
WT
RIPK3-/-
Casp-1/11-/-
Control
Immunized
e
IFN-γ producing cells/10^6 total
WT
RIPK3-/-
Casp-1/11-/-

Supplementary figure 7: Antigen-specific $CD8^+$ T cell response depends on antigen-delivery vector. (a-c) *In vivo* elimination of target cells pulsed with high (10nM), intermediate (0.1nM) and low (0.001nM) concentration of $OVA_{257\text{-}264}$ peptide at day 7 post-immunization with rhAd5-OVA. (a) Percentage of $CFSE^{High}$, $CFSE^{Low}$, CTV^{High} and CTV^{Low} shows the frequency of remaining cells after CTL-mediated target cell elimination. Percentage of live target cells in (b) non-immunized and (c) immunized mice. (d, e) Frequency of IFN-γ producing cells: Splenocytes from WT, $RIPK3^{-/-}$ and Casp-$1/11^{-/-}$ immunized or non-immunized mice pulsed with 10mM of $OVA_{257\text{-}264}$ peptide. Spots represent the frequency of OVA specific IFN-γ producing $CD8^+$ T cells. The data is expressed as means of five individual mice per group and is representative of three independent experiments with similar results.
